# *ACTA2* mutation and postpartum hemorrhage: a case report

**DOI:** 10.1186/s12881-017-0505-5

**Published:** 2017-12-04

**Authors:** Kylie Cooper, Stephen Brown

**Affiliations:** Department Obstetrics, Gynecology and Reproductive Sciences, Burlington, VT 05401 USA

**Keywords:** Aortic dissection, Postpartum hemorrhage, Smooth muscle, Genetics, Case report

## Abstract

**Background:**

*ACTA2* encodes smooth muscle specific α-actin, a critical component or the contractile complex of vascular smooth muscle. Mutations in *ACTA2* are the most common genetic cause of thoracic aortic aneurysm, and are also the cause of other disorders, including Moyamoya disease, coronary artery disease and stroke as well as Multisystemic Smooth Muscle Dysfunction Syndrome. We note that *ACTA2* is also expressed in uterine smooth muscle, and this raises the possibility that women harboring *ACTA2* mutations might exhibit uterine smooth muscle dysfunction.

**Case presentation:**

We present a young woman whose *ACTA2* mutation was ascertained during pregnancy because of her father’s history of dissecting aneurysms. She was delivered at full term by cesarean section and subsequently had severe uterine hemorrhage due to uterine atony. Although her atony was successfully treated with uterotonic medications, she required blood transfusion.

**Conclusions:**

This case raises the possibility that women with *ACTA2* mutations may be at risk of uterine muscle dysfunction and hemorrhage. Obstetricians should be alerted to and prepared for this possibility.

## Background

Thoracic aortic aneurysm with dissection (TAAD) is well known to occur in the setting of Mendelian syndromes such as Marfan and Loeys-Deitz [1]; however, non-syndromic familial dissecting thoracic aneurysm also occurs and is more common than syndromic TAAD. It is estimated that TAAD occurs in about 1% of the population and that it is familial in about 20–25% of the cases [2]. Over the past decade, there has been great progress in understanding the molecular basis of familial TAAD, with about 20 genes now known to have a causal role in affected families [3]. Familial TAAD genes fall into three major groups, those that encode various components of the transforming growth factor beta signaling cascade, and those that encode components of the smooth muscle contractile apparatus and those that encode extracellular matrix proteins. The most commonly encountered genetic cause of non-syndromic TAAD are mutations in *ACTA2*, which encodes vascular smooth muscle specific α-actin, a critical component of vascular smooth muscle contractile apparatus. The aortas of patients with *ACTA2* mutations show disorganization of smooth muscle cells of the medial layer, which is consistent with the idea that medial dysfunction is the cause of aortic dissection.

Interestingly, heterozygous missense mutations in *ACTA2* can result in a variety of other vascular diseases including strokes, early onset coronary artery disease and Moyamoya disease, indicating that structural weakness of vessels is unlikely to be the only manifestation of such mutations [4, 5]. Also, specific variants of *ACTA2* have been implicated in the Multisystemic Smooth Muscle Dysfunction Syndrome (OMIM 613834), indicating that *ACTA2* plays important roles in the smooth muscle of non-vascular tissues [6].

Maternal adaptation to pregnancy involves a multitude of complex changes in hemodynamic function such that intravascular volume and cardiac output are markedly increased. The uterine vasculature, in particular, undergoes extensive remodeling in order to accommodate the growing feto-placental unit [7]. Little is known about how *ACTA2* mutations might affect maternal physiology in pregnancy. One study examined cardiovascular complications of *ACTA2* in pregnancy and found a rate of 6% for third trimester or postpartum aortic dissection in a cohort of fifty three women either at risk of inheriting or diagnosed *ACTA2* [8]. In the cohort of thirty nine women with *ACTA2* mutation the rate of peripartum dissection was 20%, a dramatic increase above baseline peripartum risk of 0.6%. This report confirms that severe vascular complications can and do occur in the setting of *ACTA2* mutations, but does not discuss other possible pregnancy complications.

Although the most abundant and best studied myometrial actin is the gamma isoform, the gravid uterus also expresses alpha 2 smooth muscle actin [9], which raises the possibility that *ACTA2* mutations could lead to abnormal myometrial function. During pregnancy, myometrial composition, physiology and histology are continuously evolving. Actin, the main contributor to the myometrial contractile unit and component of cytoskeleton has been shown to undergo adaptations in both alpha and gamma expression in the rat [10]. Temporal changes in expression and localization likely prepares the uterus for contractions and labor. Given that alpha smooth muscle actin is expressed in uterine myocytes and is involved in uterine remodeling in pregnancy, it would not be surprising to find that *ACTA2* mutations can impact uterine muscle function.

We aim to highlight the importance of clinical evaluation, reproductive counseling and appropriate obstetrical management in reproductive aged women with *ACTA2* mutations by reporting a case of a pregnant woman whose delivery was complicated by severe postpartum hemorrhage, plausibly related to *ACTA2* mutation and uterine smooth muscle cell dysfunction.

## Case presentation

A healthy 26 year old woman presented for prenatal care with her first pregnancy. Her family history was significant for concern for Marfan Syndrome in her father due to his history of iliac artery dissection at the age of 19 followed by a thoracic aortic dissection requiring surgical repair and replacement of the aortic valve at the age of 21. He had since had another aortic valve replacement and was alive and well at the age of 53. Previous clinical genetic evaluation of both the patient and her father had determined that neither met diagnostic criteria for Marfan Syndrome. The patient’s father reported that his father died in his early 40s of a “heart attack”. He distinctly remembers that the autopsy of his father showed that the blood vessels of his heart were “filled with muscle”. None of his 4 siblings is affected with any significant heart disease or stroke. The patient herself had undergone screening echocardiograms as a child due to this family history with no abnormalities noted and no notable cardiac or vascular problems.

In light of her father’s significant vascular pathology the patient was counseled of a high likelihood for an inherited predisposition for aortic dissection. She was further counseled that the best way to pursue this possibility would be to screen her father for mutations in genes known to be involved in aortopathy. In the meantime, the patient underwent an echocardiogram at 16 weeks gestation, and this showed that her aortic root had an internal diameter of 3.7 cm (normal range 2.0–3.7 cm) and no other abnormal findings. The patient’s father agreed to testing, and a Next-Gen sequencing panel for aortopathy genes was ordered. A total of 25 genes, including, *ACTA2, CBS, COL3A1, COL5A1, COL5A2, FBN1, FBN2, FLNA, MFAP5, MED12, MYH11, MYLK, NOTCH1, PRKG1, SKI, SLC2A10, SMAD3, SMAD4, TGFB2, TGFB3, TGFBR1, TGFBR2, FOXE3, LOX, MAT2A* were sequenced.

Sequencing revealed a pathogenic variant in exon 4 of *ACTA2*, which changes Asparagine to Serine at position 117 (N117S). Amino acid residues 117 and 118 of *ACTA2* are thought to play a critical role in actin polymerization [11], and N117S has been previously reported in a family with TAAD [12]. Other families with a different substitution at the same codon (N117 T and N117I [13]) and the adjacent residue (R118Q [4]) have also been reported. Targeted analysis of the patient’s *ACTA2* gene revealed she had inherited the N117S variant from her father.

The patient’s prenatal course was uncomplicated except for a diagnosis of gestational hypertension at term for which her labor was induced at 39 + 3 weeks gestational age. Due to non-reassuring fetal assessment she underwent a low transverse cesarean section that was complicated by uterine atony. Administration of uterotonic medications improved her uterine muscle tone intraoperatively; however, in the immediate hours following delivery patient began to have heavy vaginal bleeding, likely due to ongoing uterine atony. Physical exam revealed a large, boggy and distended uterus containing approximately 1000 mL of blood clot consistent with continued atony. Management included manual evacuation of clot and additional uterotonic medication. Her total estimated blood loss was 3000 mL, which was associated with a significant drop in Hematocrit from 37 to 25%. She was transfused 3 units of packed red blood cells and subsequently recovered well with normal lochia and postpartum course.

## Discussion and conclusions

The risks of aortic dissection, coronary artery disease and stroke associated with missense mutations in *ACTA2* are well known. The case we present raises the possibility that there may also be risks of uterine atony and hemorrhage in reproductive age women.

Certainly there is both animal and human data that demonstrates uterine smooth muscle adaptation and evolution during gestation [10]. The role for gamma and alpha actin in smooth muscle function as well as their roles in the cytoskeleton and vasculogenesis is a complex interplay, one that must come together to function as a contractile unit to quickly prevent excessive blood loss. Given the potential for altered polymerization of actin, skewed ratios of isoforms and general dysfunction in the myometrium we feel that postpartum hemorrhage may in fact be a very real and dangerous complication for this specific cohort of women. Given this information and appropriate foresight providers could preemptively act to help reduce the risk for hemorrhage at the time of delivery.

Uterine smooth muscle function is critical in childbirth in preventing life threatening hemorrhage following delivery. In women who carry the *ACTA2* mutation it is essential to provide adequate and accurate counseling on risks of pregnancy ranging from life threatening aortic dissection to uterine atony and postpartum hemorrhage. Ideally this counseling would be pre-conception with a focus on informed decision making and family planning. In pregnancy, these patients warrant appropriate cardiac work-up, referral and management by high risk obstetricians to try to best minimize adverse outcomes.

## Abbreviations

TAAD: Thoracic aortic aneurysm with dissection
